# Protease-Activated Receptor 4 (PAR4): A Promising Target for Antiplatelet Therapy

**DOI:** 10.3390/ijms19020573

**Published:** 2018-02-14

**Authors:** Gamariel Rwibasira Rudinga, Ghulam Jilany Khan, Yi Kong

**Affiliations:** 1 School of Life Science & Technology, China Pharmaceutical University, 24 Tong Jia Street, Nanjing 210009, China; 2720150013@stu.cpu.edu.cn; 2 Jiangsu Center for Pharmacodynamics Research, Evaluation and Drug Screening, China Pharmaceutical University, Nanjing 210009, China; u4574904@hotmail.com

**Keywords:** protease-activated receptor 4 (PAR4), thrombin, platelet, thrombosis, PAR4 antagonist

## Abstract

Cardiovascular diseases (CVDs) are currently among the leading causes of death worldwide. Platelet aggregation is a key cellular component of arterial thrombi and major cause of CVDs. Protease-activated receptors (PARs), including PAR1, PAR2, PAR3 and PAR4, fall within a subfamily of seven-transmembrane G-protein-coupled receptors (GPCR). Human platelets express PAR1 and PAR4, which contribute to the signaling transduction processes. In association with CVDs, PAR4 not only contributes to platelet activation but also is a modulator of cellular responses that serve as hallmarks of inflammation. Although several antiplatelet drugs are available on the market, they have many side effects that limit their use. Emerging evidence shows that PAR4 targeting is a safer strategy for preventing thrombosis and consequently may improve the overall cardiac safety profile. Our present review summarizes the PAR4 structural characteristics, activation mechanism, role in the pathophysiology of diseases and understanding the association of PAR4 targeting for improved cardiac protection. Conclusively, this review highlights the importance of PAR4 antagonists and its potential utility in different CVDs.

## 1. Introduction

In the 1990s, researchers discovered PAR1 during their study of the receptor-mediating cellular actions of thrombin [1]. Thereafter, PAR2, PAR3 and even PAR4 (which is the most recently discovered in the PAR family member) were discovered [2].

PARs belong to the family of G-protein-coupled receptors (GPCR) [3], and their activation involves the proteolytic cleavage of the N-terminal sequence of PARs, that remains tethered after cleavage, and binds intra-molecularly to activate the receptor and induce intracellular signal transduction [4]. Members of this family are expressed in different cell types including immune cells, platelets, smooth muscle cells, and endothelial cells. Several studies have shown that PAR4 is highly expressed in platelets [5], lung, thyroid, testis, small intestine, and pancreas [6]. Apart from its high contribution in coagulation of blood, thrombin contributes to different biological activities, such as inflammation and wound healing [7]. Through PARs cleavage, thrombin plays a significant role in platelet activation. It is one of the platelet agonists generated by coagulation system [8]. Human platelets express PAR1 and PAR4, and studies have demonstrated that their activation may promote platelet aggregation and secretions [7].

It has been recognized that platelets contain different biological substances which can be released anytime when they are activated. Ma et al. (2005) [7] have reported that platelets in the blood from rats contain endostatin, a potent inhibitor of angiogenesis and is released in response to PAR4 in an aggregation-independent manner. In their study, they have also demonstrated that human platelets contain endostatin, and that its release can be only triggered by PAR4 activation [7].

Different studies have shown that Leucine 43 at position 5 on PAR4 may facilitate the binding and cleavage of alpha-thrombin [9] to the PAR4 N-terminal site [8]. In the analytical study using NMR on PAR4 binding to thrombin, Cleary et al. (2002) [10] reported that less contact is made by the Leu43 at the P5 position and it exhibits two conformational states (Leu43i and Leu 43ii). Their study showed that there is a certain degree of flexibility in that Leu43 of PAR4 helps it to interact with the thrombin. However, after observing their NMR results on a PAR4 peptide they suggested that the P4–P1 positions dominate in anchoring this peptide onto the thrombin surface, and due to the flexibility observed in solution for the Leu43 position, the Pro44 was proposed to be a more important player in the binding of PAR4 to thrombin [10]. Besides the fact that thrombin interacts with both PAR1 and PAR4 however, the resultant action is somehow different. PAR4 does not contain a hirudin-like sequence (K51YEPF55) as it appears on PAR1, but investigations show that PAR4 interact with thrombin through Proline44 and Proline46, which are efficient positions for PAR4 cleavage by thrombin [8]. Compared to PAR1, PAR4 provides slow Ca^2+^ signalling, and this slow signaling leads to slow cleavage by thrombin resulting in a sustainable platelet aggregation [11]. Nieman. et al. (2007) [12] have reported that leucine and proline amino-acids of PAR4, are synchronized which collaborate to build a 3-dimensional structure which is efficient and can easily facilitate thrombin in the cleavage process [12]. Similarly, another study by Jacques and Kuliopulos identified the easiest way of interaction between α-thrombin and PAR4 via prolines at position 4 and 2 [11]. Aggregately, these studies indicate that specific PAR4 positions (P4 and P2) may provide essential ways or a strategy for its targeting and serve in the development of PAR4 thrombin receptor antagonists.

Thrombin or factor Xa which is able to cause PAR4 cleavage, represent the most critical factor of coagulation in human blood. However, Xu et al. (1998) reported that there is a limited cooperation between PAR4 and thrombin via its exosite-I part and the exosite-I mutations have less effects on PAR4 cleavage compared to PAR1 [5,13]. Activation of PAR4 through proteolytic cleavage takes place after the change and removal of the N-terminal exodomain of PAR4 to the new amino terminus named tethered ligand [4] and this will promote activation of G protein coupling and multiple intracellular signaling [14]. During the internalization process, Gq, and G12/13 play a vital role to coordinate signals provided by PAR4 activation.

Studies identified that PAR4 activation mediates intracellular signaling via the G-protein subunits Gq and G12/13, but not via the Gi signaling pathway [15,16]. By coupling to G12/13, PAR4 activates Rho GEF that will induce the phospholipase C (PLC) activation and triggers the change of platelet shape which is an important key in platelet aggregation during thrombosis [13]. Whereas, coupling to Gq induces calcium mobilization through activation of PLC-β and triggers kinase and phosphatase activation which promote several key platelet responses (integrin activation and release of stored granules) contributing effectively in platelet aggregation. These aggregated platelets are the causal agents of thrombosis in human blood. They form what can be called a platelet plug and activate the release of agonist substances involved in recruiting many other platelets in circulation [6].

## 2. PAR4 Structural Features, Activation Mechanisms, and Signaling

**Figure 1:**
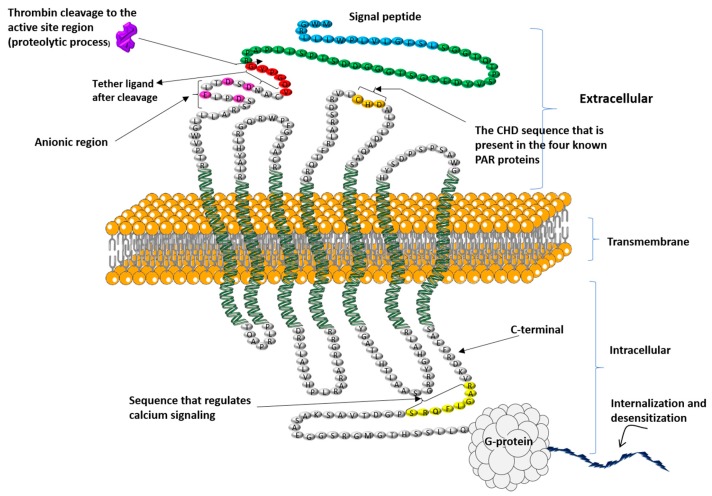
The proposed structure of human PAR4 with its different parts and their functions. Thrombin cleavage site that reveals a neo-amino-terminus (showed by arrow), the tethered ligand sequence that binds to the second intracellular loop after thrombin cleavage (sequence with the red color), and the Cysteine, Histidine, Aspartic Acid (CHD) amino acids sequence that is present in the four known PAR proteins (gold color), the RAGLFQRS sequence (showed by yellow color) discovered by Ramachandran and colleagues [17] for its role in regulating calcium signaling and interactions of the receptor with β-arrestins (1 and 2), and the anionic region that play a role of interacting with thrombin exosite-I (showed by rose color), and the signal peptide (showed by blue color), the amino-terminal peptide cleaved by thrombin (showed by green color), then the remaining extracellular and intracellular regions are shown in gray.

Structurally, PAR4 is made up of 300 amino acids and is a member of the GPCRs family. It is the channel used by different proteolytic enzymes to initiate internal signaling during the platelet activation process. It contains different parts reserved for specific roles, such as a thrombin cleavage site, reserved for proteolytic cleavage of the receptor N-terminal, the tethered ligand sequence formed after cleavage reveals a neo-amino-terminus. The binding regions for the newly formed tethered ligand binding site falls at the second extracellular loop of the receptor, and the anionic part which interacts with exosite-I of thrombin [13] (Figure 1).

The study done by Ramachandran and colleagues (2017) [17], identified an intracellular C-terminal sequence in PAR4 (RAGLFQRS) which regulates calcium signaling and is critical for PAR4-β-arrestin interactions (Figure 1). Thus, they reported that the receptor missing this domain was nonetheless able to trigger activation of MAP kinase. This has been confirmed by their study demonstrating that by targeting this sequence with a cell-penetrating peptide (RAG-8). Eventually, they were able to block PAR4-dependent platelet activation in vitro and attenuate clot stabilization in vivo without affecting signaling by PAR1. Their study also demonstrated that removal of a C-terminal motif disrupts Gq dependent calcium signaling [17]. However, further studies are needed for the in-depth understanding of this mechanisms of inhibition.

The work of Nieman’s group identified that the anionic group of the N-terminal part of PAR4 is essential for interaction with α-thrombin. Another study by Cantellas et al. (2017) [18] on the extracellular loop II of PAR 4, concluded that PAR4 contains a cluster of electronegative residues in the extracellular loop II (ECLII), with aspartic residues in positions 224, 230 and 235, and another cluster at positions 57, 59 and 66 in the N-terminal, which are important for PAR4 binding to thrombin. They further suggested that these positions play their characteristic roles for the tethered ligand binding to the receptor. In the ECLII, PAR4 cleavage is facilitated by interactions between α-thrombin and the aspartic residues, which means that ECLII of PAR4 could be an influential target for antithrombotic drug development [18].

The activation mechanism of PAR4 is initiated by a ligand that switches from the stable N-terminal part of the receptor, activates it and induce its conformational changes [8] that facilitate its binding to the second extracellular loop and enhance the activation leading to intracellular signal transduction [4]. PAR4 contains an important cleavage site at Arg47/Gly48 position on the N-terminal part which facilitates thrombin cleavage and receptor activation [19]. It is the most important site for thrombin cleavage because the mutation of Arg47→Ala sequence at this site was shown to prevent proteolytic activation of the receptor [13]. Indeed, leucine 43 of PAR4 showed its big role in the interaction between thrombin and receptor, by inducing the binding of PAR4 N-terminal to thrombin’s active site. Studies have shown that this Leu43 has specific interactions with particular amino-acids from thrombin (Leu99, Ile174, and Trp215) which help in thrombin cleavage during PAR4 activation [12]. Further studies on PAR4-thrombin interaction demonstrated that there is an attraction mechanism between PAR4 anionic [20] clusters (Asp57…Asp59…Glu62…Asp65) and cationic residues bordering exosite-I of thrombin, which easily facilitates interaction between them and contributes to a lowest dissociation rate of thrombin from PAR4. An interesting study on the interaction of four receptors with α-thrombin by Nieman et al. (2007) reported that leucine43 and proline44 at P4 are essential for thrombin cleavage [12].

However, signaling rate between PAR1 and PAR4 is different even if both are activated by thrombin [2]. Nieman et al. recognized that one of the reasons for this difference in signaling is the length of PAR4 exodomain which is shorter compared to PAR1 exodomain (62 amino acids for PAR4 versus 75 amino acids for PAR1), which make the PAR4 cleavage site closer to the plasma membrane, and ineffective to activate compared to PAR1 [12].

**Figure 2:**
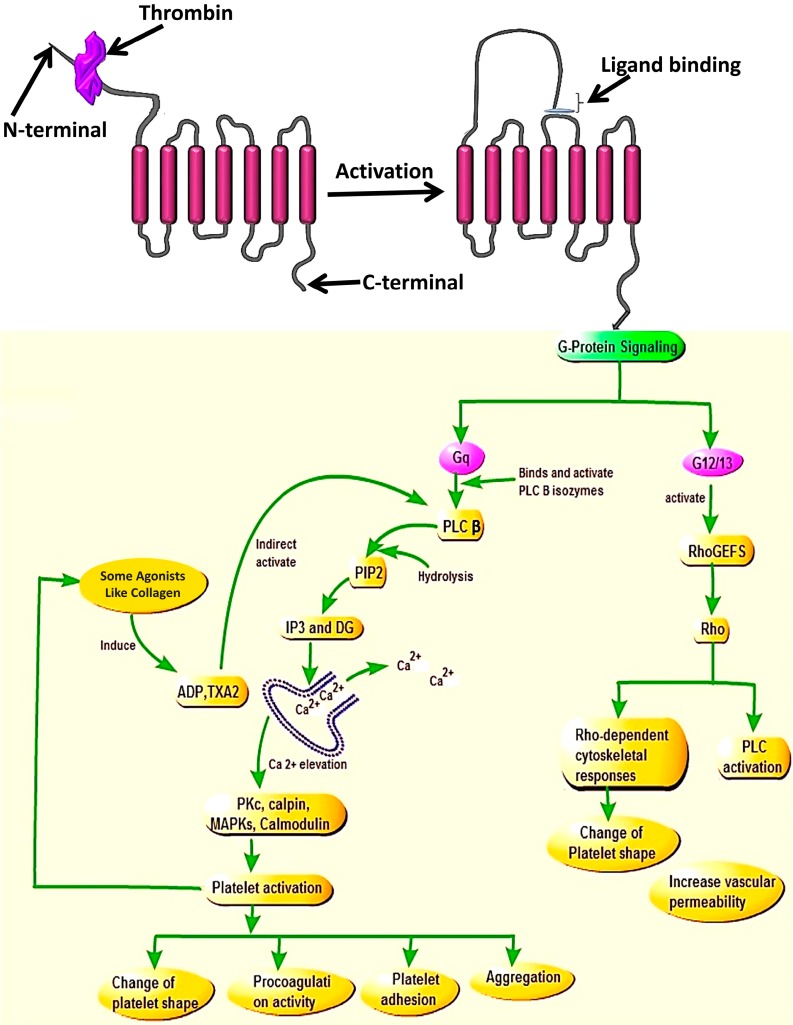
PAR4 signaling mechanism via Gq or G12/13 family members after cleavage of receptor.

Once cleaved and activated, PAR4 is internalized on a phosphorylation signal through the C-terminus site of the receptor. Two types of G-protein subunits (Gq and G12/13) assist activated PAR4 to coordinate intracellular signaling during the internalization process [16]. However, binding of PAR4 to these two G-protein subunits led to the release of different substances. Upon binding with G12/13, PAR4 interacts with some exchange factors (like FXI), the action which stimulates the functioning of the PLC enzymes and ultimately leads to platelet shape change, which is the hallmark of initiation of platelet aggregation during thrombosis [21,22]. Whereas, when PAR4 binds to Gq [23] it activates the release of PLC [24] which contributes in the hydrolysis of membrane phosphoinositide to releasing inositol triphosphate (IP3) and diacylglycerol (DAG) [23] that will stimulate endoplasmic reticulum to mobilize Ca^2+^ and activation of internal protein kinase C (PKC), known for its role in the protein phosphorylation. The combination of all these processes plays a central role in platelet activation and recruitment of other platelets in the blood circulation to attach and change platelet shape [4,13] (Figure 2). Studies showed that once activated by a protease, the PARs need to be turned off to prevent indefinite signaling. These (PARs) will be also replaced in order to permit a second round of responses to the protease, and the way this is accomplished varies among family members and in different cells [25]. Then, following internalization, PARs will be sorted for either degradation (lysosomes) or recycling. While on another side activated receptors are predominately packaged into lysosomes for complete signal termination. For PAR4 specifically, the study showed that its additional membrane trafficking pathway is provided by efficient transport from the endoplasmic reticulum (ER), via the Golgi for post-translational modifications such as glycosylation [26]. There are several mechanisms for terminating signaling through other G protein coupled receptors that also apply to PAR family members. These include receptor desensitization (done by uncouples receptors from their G proteins), receptor endocytosis (removes cleaved receptors from the cell surface), and receptor down-regulation that leads to a reduction in total receptor number [27].

Indeed, studies have shown that calcium mobilization can be promoted by activated P2Y1 receptor bound by ADP, the process that will initiates the Gq/PLCβ signaling pathway, and provide IP3 after hydrolysis of certain phosphates, and affect stored calcium to be elevated and then mobilized, which will also assist in platelet aggregation [6].

The N-terminal and activation of the receptor allows the tethered ligand to bind to the second extracellular loop and the signaling downstream of Gq contributes to Ca^2+^ mobilization and elevation via PLCβ activation, which will lead to the activation of the kinases and phosphatase (MAP Kinases, PKC, Colmodulin, Calpin) resulting in platelet activation and cellular responses such as change of platelet shape, procoagulation activity, platelet adhesion, and aggregation. While on the other side, the signaling of G12/13 via RhoGEfs will cause the activation of Rho and also promote activities of Rho-kinase and PLC which will cause the change in platelet shape and increase vascular permeability.

Covic et al. showed that PAR4 coupling to Gq elicits a slower stimulation of intracellular calcium mobilization compared with PAR1 which stimulates a rapid increase in intracellular calcium, and this prolonged signal from PAR4 activation is more sustained over time and has been shown to be necessary for stable clot formation [8]. For all PARs, normally, their complete signal termination depends on the way they are internalized, however, studies have demonstrated that after signaling, they are degraded by lysosomes, incidentally showing unique characteristics of each receptor; there are no perceptible methods to identify how this degradation process is done [4].

## 3. PAR4 Co-Factors and Heterodimerization with Other Receptors

In several instances, the dimerization mechanism of the PAR has been realized as the special mechanism promoting the activation of an adjacent PAR [21]. Different studies have shown that PAR4 forms heterodimers with other receptors, especially, it forms a stable heterodimer with PAR1 [13]. Normally, when these two receptors are co-expressed, it is difficult to comprehend their difference of affinity to thrombin, because of heterodimerization between them and many studies suggested that the formed heterodimerization is critical for platelet activation [28]. PAR4 contributes to the signal transduction process, but itself can be controlled by interactions with other receptors, where some of them may work like cofactors or for dimerization [29].

However, the complex formed by PAR1/PAR4 is critical for proper platelet activation by thrombin because PAR-1 acts as a co-factor for PAR-4 activation when the thrombin is still bound to PAR-1, the action that causes PAR-4 to be activated and triggers a prolonged Gq signal which will make the aggregation to become irreversible. This action shows that PAR4 acts as an ancillary receptor for PAR1 [30]. The study showed that thrombin activation of PAR1 results in the quick increase in intracellular Ca^2+^ which is directly followed by PAR4-mediated sustained Ca^2+^ response. Shortly, the activation of PAR4 by thrombin appeared more effective at sustaining the secondary signaling responses, a process important for the late phase of platelet aggregation [31]. This means that PAR1 is responsible for triggering the initial spike of intracellular calcium but PAR4 is acting as an ancillary receptor to initiate a second calcium wave which will stay for a long time.

Nakanishi et al. (2000) show that the co-factoring and dimerization mechanism of PARs intervene in efficient G protein activation, enhancing its coupling specificity to accelerate coupling to distinct effectors [32]. Different studies showed that PAR3 and PAR4 are co-expressed in mouse platelets [33]. However, the heterodimers between PAR3 and PAR4 have not yet been reported. PAR4 forms a stable heterodimer with PAR1, the mechanism that enables thrombin to activate both receptors, which will cause a prolonged Gq signal and make the aggregation sustainable and irreversible [34,35].

After its cleavage by thrombin, PAR1 becomes a co-factor, and arranges its hirudin-like region to remain bound to thrombin exosite-I for protease conformation. Thus, at that time, an adjacent PAR4 will interact easily with thrombin through its anionic region and make the thrombin cleavage site well activated [6]. Several published studies now indicate that PAR1 plays an important role in PAR4 activation by thrombin, and they form a complex heterodimerization in human platelets [34,36]. Furthermore, studies reported that PAR4 has direct and indirect effects on P2Y12 signaling in platelets. Following activation, PAR4 and P2Y12 form heterodimers and functional co-cooperativity to recruit arrestin-2 to mediate PI3K-dependent AKT activation that promotes platelet activation and stabilizes thrombi. In human platelets, it has been shown that PAR4 works in synergy with the P2Y12 receptor to induce platelet activation [6,37]. In addition to that, PI3K has been found to be involved in several other pathways [38].

PAR4 also interacts with the α2A-adrenoceptor, and this cooperation has been identified to alter the human platelets which used aspirin during their treatment through interactive signaling [13]. Indeed, PAR4 interacts with bradykinin B2 receptor, which assists pro-inflammatory effects. So, all of these mentioned receptor dimerizations show how PAR4 signaling is complex and the way it is capable of inducing several pathways to cause physiological responses, and it has been reported that PAR possibility of co-factoring and heterodimirization with other PARs requires that two of them must be close to each other [33].

## 4. Targeting and/or Inhibition of PAR4 Activation, a Considerable Therapeutic Value

Different studies demonstrated that activation of PAR4 by thrombin on the platelet surface is linked to many responses essential to hemostasis. The activated platelet causes rapid intracellular calcium mobilization, secretion of autocrine hormones, production of eicosanoids such as thromboxane, and raising expression of some glycoproteins which are important in the interaction of platelets with hematopoetic cells, implicated in coagulation and inflammation [39]. Compared with other PAR family proteins, activation of PAR4 will depend on the increased concentration of thrombin [40]; its study in platelets demonstrated its big physiologic function in different diseases. Mumaw. et al. (2015) demonstrated that targeting PAR4 can protect myocardial ischemia [41]. Through their study done in rodents, they also showed that PAR4 assists in joint pain and inflammation and plays a role in diabetic vasculopathy. Thus, according to these different studies, it is clear that PAR4 plays a substantial role in multiple pathophysiological settings, and its targeted antagonists will be of consideration to therapeutic value [2]. Indeed, its inhibition may be one of the best strategies to inhibit PAR1 activity and decrease the prolonged signals triggered by thrombin through PAR1 mediation which activate close PAR4 [42]. That is why deep understanding of the therapeutic value of targeting PAR4 and development of its effective pharmacological inhibitors is an important thing because nowadays it is still on a lower level compared with other therapeutic agents developed for targeting other PARs that may be associated with limited scientific knowledge about PAR4 [33,43].

## 5. PAR4 Agonists

Today, the PAR4 agonists are designed peptides according to the sequence of the tethered ligands of the receptor. The agonists including GYPGKF-NH2, AYPGKF-NH2, and GYPGQV-NH2 are selectively activating PAR4 [13].

## 6. PAR4 Antagonist

The aim of different researchers into the role played by PAR4 in different angles, emphasizing the research on the specific and convenient PAR4 inhibitor. However, some particular challenges may prevent the development of real inhibitors. Specifically, targeting PAR4 activation by focusing on the proteolytic cleavage mechanism is not an easy strategy due to the irreversibility of the system, and on the other hand, the inhibition of signal transduction generated by newly formed tethered ligand; there is also a need for specific antagonists that can bind to agonists intrinsically and prevent trafficking mechanisms [13]. Indeed, there are many differences in the PAR4 structure that differentiate it from other receptors of the family. An example, both the extracellular amino-terminus and intracellular carboxy terminus have little sequence similarity to the corresponding regions of other PARs [5]. PAR4 also lacks the high affinity thrombin binding domain that is present in the other thrombin receptors (PAR1 and PAR3) [44].

## 7. PAR4 Implications in Various Cellular Responses

In a study on isolation of lungs from mice, Peters and Henry reported that PARs are needed for respiration and react to infection in the body. They showed that PR is more important in raising the permeability capacity of thrombin in the pulmonary small vessels which decrease their size. Specifically, studies have demonstrated that the highest PAR4 neuronal expression enhances brain injury in rat models, showing its role in destroying neurons results in ischemic adaptation [4].

Normally, it is unknown whether human platelets possess endostatin or not. Similarly, the relationship between PAR4 and PAR1 in controlling endostatin secretion is also unknown. However, what is known is that this endostatin is the most potent anti-angiogenic factor which can retard or prevent angiogenesis. Researchers discovered endostatin in human platelets, and it has been reported that PAR4 [45] activation assists endostatin release, leading to angiogenesis impairing and wound healing [33]. It has also been proposed in earlier studies that PAR4 possess some role in embryogenesis [46]. Studies identified that when the formation of the platelet labyrinth layer is disturbed during early placental morphogenesis, it may affect a pregnant woman by losing her fetus.

Through their study on mice, Sood and colleagues [47] identified that activated PAR4 assists in pregnancy failure via its implication in the maternal platelets. Additionally, several recent studies on PAR4 implication in embryogenesis identified that inhibition of activated PAR4 may partially protect fetal loss and stabilizes placental morphogenesis [47]. It has been identified that PARs, in general, are implicated in the activation of different cytokines in the cell, and mediate responses that mainly lead to inflammation [48,49], pain, swelling, and redness. The study done on rat cortex using PAR4 activating peptide showed that this PAR4 agonist (AP GYPGKF) raises the TNF-α expression that enhances inflammation in human cells. In addition to their pathological roles, PARs also have been recorded in mediating cancer progression [9]. Several current studies identified that expression of PAR4 is highest in prostate cancer, and it also mediates the migration of colon cancer and hepatocellular carcinoma-derived cell lines [4]. However, in the cancer targeting strategy, some researchers use platelets due to their high interaction with circulating tumor cells and their ability of carrying and prolonging cancer drugs during their circulation, where the drugs are moving around in the body but protected from the immune system, allowing them to stay for a long time in the blood [45].

## 8. Effects of PARs on Various Aspects of CVDs

All the PAR family members are normally expressed in endothelial cells (arterial or venous). However, their level of expression is different. It was shown that in both mice and humans, PAR1 is the main mediator of thrombin signaling in vascular endothelial cells. The study by Johnson et al. (1998) [50] demonstrated that activated PAR1 by thrombin triggers endothelial cell contraction, which affects the cell to cell interaction and provides an easy way of molecules and cells from the blood to occupy sub-endothelial gaps and help in exposure of tissue factors and collagen. Furthermore, studies showed that platelet activation mediated by PAR1 or PAR4 signaling allow the attachment to the atherosclerotic lesion, facilitating an easy reactive area for the recruitment of monocytes and lymphocytes [50]. Platelet activation and aggregation will result in the formation of platelet thrombi, the latter will be precipitated and then may occlude the arteries, leading to acute ischemic events such as acute coronary syndromes, stroke, and transient ischemic attack. PAR2 is implicated in the arterial and venous dilatation in vivo in healthy humans. Indeed, researchers have shown that PAR2 activation could contribute to blood pressure dysregulation during sepsis, endotoxemia, or other states associated with serine protease activation [51].

Cardiomyocyte cells expressed PAR1 and PAR2 [52]. The study showed that PAR1 and PAR2 activation in the ventricular myocytes, strongly activates phosphoinositide hydrolysis, induces the extracellular protein kinase, triggers atrial natriuretic factor expression, and activates calcium homeostasis [53]. All of these mentioned events significantly change the electrophysiologic properties and contractile behavior leading to cardiomyocyte hypertrophy. However, despite cardiomyocyte cells, several studies have shown that PAR activation induces vascular smooth muscle proliferation, migration, and collagen synthesis leading to plaque progression, which may play an important role under pathologic conditions such as atherosclerosis [54].

## 9. Pharmacology

Nowadays, there are different types of antiplatelet drugs which are clinically used as a therapeutic measure to fight against thrombotic disorders. The core focus of researchers which provide these drugs is to target and/or inhibit thrombin receptors (PARs) for developing a better antithrombotic therapy. They generally focus on targeting agonist effects or its generation [6], and different coagulation cascades which enhance thrombin activity [8]. For PAR4 particularly, there are many anti-PAR4 inhibitors that have been developed, but only a few of them have been studied in clinical settings [34]. Moreover, several new drugs have been reported for antithrombotic actions [55] that may further be explored in this direction.

Earlier studies have reported that human platelets express PAR1 and PAR4. However, studies demonstrated that inhibition of PAR1 as an antiplatelet function can only provide a partial impact on the thrombin potential to control the activation of platelets. That is why many studies concluded that combination of PAR4 and PAR1 targeting could be an effective method of antithrombotic effects [2]. Until now, there are no inhibitors, or combination of these drugs (against PAR1 and PAR4) that have successfully shown a perfect clinical improvement without any side effects. These challenges brought the development of different PAR4 antagonists, which nowadays have started to overcome this issue, by different antagonist classes. These antagonists are function blocking antibodies, peptidomimetics, and low MW compounds [34].

## 10. Function-Blocking Antibodies

An antibody may function as a PAR antagonist compound when it can block cleavage-activation sequences of a PAR and unmask its tethered ligand sequence [2]. Today, there are different function-blocking antibodies specifically against PAR4, and therapeutically, they have been proven to be antagonists against PAR4 (Table 1). A rabbit polyclonal antibody designed to target PAR4 was shown to be an important function-blocking antibody [33] used particularly to block the PAR4 thrombin cleavage site in human [56], hence prevent aggregation of human platelets measured at 1 mg·mL^−1^ [13]. Mumaw and colleagues [57] developed an anti-PAR4 antibody directed toward the sequence C54ANDSDTLELPD of the anionic region of PAR4 (an essential region where PAR4 interact with thrombin) using purified exodomains and cell lines. Interestingly, the developed antibody (named CAN12) has been identified for anti PAR4 activation by interrupting and blocking the interaction between PAR4 and thrombin which reduces the rate of PAR4 cleavage by thrombin. Out of this, CAN12 also showed its strong capacity by inhibiting PAR4-activating peptide, ADP, and collagen which are all inducers of platelets aggregation in humans [57].

Wong et al. (2017) [37] developed a polyclonal antibody from rabbits and used it to target the thrombin cleavage site of PAR4 in guinea pig by just checking the capacity of antibody and their antithrombotic effects and examining how they react to the bleeding activity, and so on. Interestingly, this antibody showed effective antithrombotic capacity by lowering bleeding risk compared to clopidogrel [37].

By testing this, they evaluated anti-PAR4 antibodies bleeding risk (3.4 mg/kg of dose) in the guinea pig and checked the bleeding time models, followed by comparing the designed antibody with standard reference antiplatelet drugs (3.4 mg/kg of dose) IgG, clopidogrel or argatroban. Their results showed that anti-PAR4 antibody decreased BT compared with the mentioned standard reference antiplatelet drugs that showed increased BT [37]. These results are proof to show that PAR4 inhibition can give an effective antithrombotic effect by just decreasing the bleeding risk. Also, Mumaw et al. (2015) [41] developed monoclonal antibodies (2D6, 14H6, 5F10) purified from hybridoma producing antibodies, and two of them (14H6, 5 F10) showed to be sensitive to the activation of PAR4, and also they partially inhibited PAR4 cleavage site by α-thrombin. This means that these antibodies provide an opportunity to control endogenous PAR4 expression [41]. Hamilton al. (2017) reported that the antibody inhibited thrombin cleavage of PAR4 in transfected Rat1 cells, abolished PAR4-mediated calcium signaling in mouse lung fibroblasts, and impaired thrombin-induced human platelet aggregation in the presence of concomitant PAR1 inhibition albeit at high concentrations [58].

Nowadays, many different function-blocking antibodies against PAR4 that are used to target its different sites, combined with series of mouse monoclonal antibodies have been developed by different researchers. However, there is still a problem in targeting because some of the anti-PAR4 antibodies showed lack of specificity in their targeting where you will not only inhibit thrombin but also will inhibit the action of some other receptors like, P2Y12 and GPVI, which are not the basic targets. Others also showed to have limited capacity, like some of the series of monoclonal anti-PAR4 antibodies mentioned above designed to target anionic region sites of PAR4; they only react partially in cell activity. Despite all of these successful approaches, only very recently the utility of function-blocking anti-PAR4 antibodies have been used experimentally, and its experimental essay for targeting the thrombin cleavage site of PAR4 was highly specific and surprisingly efficacious when examined in similar platelet-based functional assays and in the in vitro human thrombosis model. However, whether or not targeting PAR4 using a function-blocking antibody-based approach in clinical studies is still in developmental stages as until now only the in vitro and in vivo animal studies have been reported while to the best of our knowledge, no human studies are available in this regard to date. Considering this aspect, we have highlighted this feature and pointed out that this direction may further be explored [33].

## 11. Peptidomimetics

Few compounds have been identified that can target PAR4. The peptidomimetic has been changed by researchers by adding a *trans*-cinnamoyl (tc) group [2]. Then, the designed combination of peptidomimetic and *trans*-cinnamoyl group (tc-Tyr-Pro-Gly-Lys-Phe-NH2) has shown the capacity of blocking aggregation of platelet enhanced by PAR4-AP and thrombin [59] in rodents, and in human platelets [13]. Bradykinin breakdown product inhibited PAR4 activation. The study done showed that this peptide inhibited platelet aggregation in humans but also showed its strong inhibition in murine platelets, where it binds to the amino-terminal part of PAR4 and prevents cleavage of thrombin to PAR4 [4].

The tcYNH2 mentioned above has been realized as the inhibitor of thrombin in human blood [33]. Currently, there are two types of peptidomimetics developed and used in different studies. Those are *trans*-cinnamoyl-YPGKF-NH2 and *trans*-cinnamoyl-APGKF-NH2 [34]. However, studies showed that this inhibitor (peptidomimetic) still has limited capacity to be used as an effective inhibitor of human PAR4 [60].

## 12. Low MW Compounds (YD-3)

**Figure 3:**
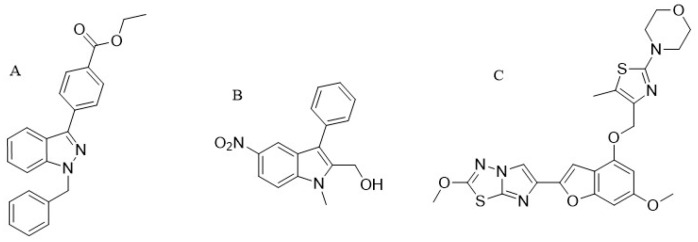
Low molecular weight PAR4 antagonists. (**A**) YD-3 [1-benzyl-3(ethoxycarbonylphenyl)-indazole], PubChem CID (10132921); (B) ML354 (1-methyl-5-nitro-3-phenyl-1H-indole-2-methanol), PubChem CID (752812); (C) BMS-986120 4-(4-(((6-methoxy-2-(2-methoxyimidazo[2,1-b][1,3,4]thiadiazol-6-yl)benzofuran-4-yl)oxy)methyl)-5-methylthiazol-20yl)morpholine, PubChem CAS (1478712-37-6). NO_2_: Nitro group.

YD-3 (1-benzyl-3 ethoxy carbonyl phenyl-indazole) (Figure 3A) was identified for the first time in 2002 as a low-molecular-weight antagonist of PAR4. Since this compound started to be used experimentally, it showed that it is a specific and a tool compound for targeting PAR4 [14]. Different studies done by testing YD-3 inhibitory activity in human platelets demonstrated that the compound inhibits aggregation of platelets triggered by the PAR4 agonist, however, little work regarding this compound has been published. YD-3 showed a selective inhibition against PAR4, after testing its antagonist activity on human platelets that express both PAR1 and PAR4 it only inhibited platelet aggregation induced by the PAR4 agonist. YD-3 inhibited PAR4-AP-induced human platelet aggregation with IC50 value 0.13 μM [61] and at the concentration of 10 mg/kg by per oral (P.O.) impaired neointima formation in rats in vivo [62]. However, this compound did not inhibit thrombin activities [2]. The same results reported by Wu et al. (2006) in their study on YD-3 inhibitory capacity in the washed human platelets with different agonists, they reported that YD-3 did not inhibit thrombin activities nor SFLLRN, collagen or U46619, but, it selectively prevented GYPGKF-intracellular calcium mobilization, one of the known important steps for activation of G-protein receptor [63]. Shortly, their data indicated that YD-3 is an effective antagonist of thrombin in mouse platelets by blockade of PAR4. Alternatively, Jang et al. (2009) [14] showed that YD-3 was capable of inhibiting purified cathepsin G-induced human platelet aggregation. This cathepsin G released from activated neutrophils has been declared as an essential mediator of cell-to-cell interactions [61]. As a conclusion to their report, YD-3 is not an inhibitor of thrombin inducing PAR4 cleavage as it cannot even prevent fibrinogen assisting in blood clotting that is empowered by thrombin. It is only in the washed human platelets, for the small concentration of thrombin where YD-3 showed a partial inhibition [64,65].

Studies have reported that YD-3 enhances conformational changes in PAR4. These changes are directly regulated by the amino acid at residue 120. In addition to this, the study showed that YD-3 could be more effective to white people compared to black ones. However, the YD-3 in vivo study showed that the usage of this small molecule still is limited probably due to its high lipophilicity [42].

ML354: The ML354 known as (1-methyl-5-nitro-3-phenyl-1*H*-indole-2-methanol) (Figure 3B) is one of the current discovered types of indole [66] that contain 282 Da of molecular weight. This compound showed that with the IC50 value of 140 nM it inhibited PAR4-AP-induced human platelet aggregation and proved to be a specific selective inhibitor for PAR4 [33,39].

## 13. Pepducins

Pepducins are lipidated peptides which are easily permeable to the cell membrane [67], designed according to their nature as an agonist to activate receptors or antagonist for blocking the interaction between the receptor and G protein by just preventing internalization of the signal in the intracellular part [13] (Table 1). Like other inhibitors mentioned above, pepducins antagonists were also identified as an inhibitor of platelet activation by blocking PAR4 and PAR1 activities. Specifically, those pepducins with a sequence corresponding to the third intracellular loop of the receptor, the site known to interact with G proteins and facilitate downstream signaling.

These pepducins are designed in the biological way where they are combined with N-terminal palmitate (pal) which facilitate them to pass through the lipidated membrane of the cell and bind to G proteins preventing internalization process [67]. The palmitoylated peptides mentioned above can inhibit or activate downstream signaling; it depends on the design of researcher. An anti-PAR4 (pal) pepducin, known as P4pal-10 (*N*-pal-SGRRYGHALR-NH2) has been identified as an antiplatelet aggregation as it inhibited PAR4-AP-induced human platelet aggregation with IC50 value 1 μM, and at the concentration of 3 μM increased tail bleeding time in mice in vivo [68].

The pepducin inhibitory activity is not only applied to PAR4 inhibition; studies demonstrated that it can also be applied for targeting platelet activation induced by PAR1 and GPVI receptors. The ability to inhibit PAR1 and PAR4 receptors is probably facilitated by the similarity of the C-terminal sequence between human PAR1 and PAR4 third intracellular loop [2,4]. By targeting different intracellular regions of the receptor, subsequent pepducin P4pal-i1 (*N*-pal-ATGAPRLPST-NH2) also has been synthesized to target the first intracellular loop (ICL) of the receptor. P4pal-il inhibited PAR4-AP-induced human platelet aggregation with an IC50 value of 0.6 μM and at the concentration of 0.13 mg/kg decreased occlusion time after thrombotic injury non significantly in guinea pigs [36]. This antagonist has been identified as a selective inhibitor of PAR4-Activating Peptides (AP) without affecting PAR1-AP induced platelet aggregation [13] and extracellular side of the receptors [68]. This can be a targeting strategy for disrupting the dimerization mechanism where a receptor provides its intracellular loops to another receptor.

Due to their ability to pass through the plasma membrane and achieve their targeting goals, pepducins have been openly used in different membrane proteins during different studies [2]. However, even if they demonstrated effective activities against thrombin-mediating platelet aggregation, these inhibitors (pepducins) still lack the specificity of anti-PAR4 targeting which is their main obstacle until today [33].

## 14. BMS-986120 (a Potent, Selective Antagonist for PAR4 Targeting)

In the identification of the small-molecule PAR4 antagonists, screening of different compounds has been performed by researchers. The compound by Bristol–Myers Squibb (BMS) named “BMS-986120” (Figure 3C) showed that is an effective and selective antagonist against PAR4 receptor.

After their strong work in the screening of 1.1 million compounds of BMS-986120 and performing their deep study, Wong and colleagues (2017) [37] reported that BMS-986120 completely antagonized PAR4 AP-induced signaling modulated through several channels, and studies in monkeys and human trials showed that the compound decreased at least thrombus formation by 82%, and it potentially inhibited vascular occlusion in all tested animals [37].

Indeed, the study identified that at the concentration of 1 mg/kg BMS-986120 decreased thrombus weight by 83% in monkey and increased BTs by about two fold compared to clopidogrel in vivo [37].

Compared with other current antithrombotic inhibitors (clopidogrel, YD3, ML354), BMS-986120 showed that it is a potent and selective antithrombotic drug for PAR4 receptor targeting. BMS-986120 has been identified as the leading compound from imidazothiazole derivatives and it was already evaluated in Phase I clinical trials in the search of an effective anti-thrombotic drug [33,57].

## 15. PAR4, a Predictor Answer for Therapy

Antiplatelet drugs, especially receptor antagonists for decreasing atherothrombotic events, are available on the market. However, there are many drawbacks and side effects that limit their use [37,70]. Recent survey reports have demonstrated that 17 million deaths are caused by cardiovascular events every year, and among these, 7.3 million are caused by ischemic heart disease and 6.2 million of deaths are caused by strokes [70]. Today, the CVDs are the leading cause of deaths worldwide [58,67]. Aggregation of platelets is the key cellular component of arterial thrombi, and different lines of evidence showed that PAR4 mediates thrombin activities enhancing activation of human platelet [41]. Thus, development of the robust drug with antithrombotic activity and lower bleeding risk is needed to fight against the disease with improved safety.

Wong and colleagues [37] studied PAR4 blockade and recognized that when PAR1 is activated, it induces the production of small concentration of thrombin to activate other platelets which is the main basic step for hemostasis. The progressive step for platelet activation with the highest concentration of thrombin (that leads to occlusive thrombosis) is mediated by PAR4. Development of PAR4 antagonist while preserving PAR1 signaling could be the safer and effective strategy to prevent thrombotic diseases while controlling hemostasis [28,37].

Different studies have shown that inhibition of PAR4 activation is the main key in restricting the pro-coagulant activity. Indeed, studies reported that PAR4 inhibition is negatively affecting prolonged calcium signal and other pro-coagulant effects that are hallmarks of thrombus formation [56]. Nowadays, different PAR4 antagonists have been discovered for targeting different sites of the PAR4 receptor. However, these agents still have different problems like lack of specificity, bleeding risks, etc. [71,72]. Given the role of PARs in general for platelet activation, PAR4 contributes to other different roles beyond platelets and has also been shown to play at least one important role to enhance platelet activation that cannot be performed by PAR1, which makes it an important novel target for fighting against thrombosis [56].

## 16. Platelet Reactivity and Racial Disparity

There are many factors that may encourage the incidence of cardiovascular disease, some of them we can say like genetic polymorphisms, medical adherence, and environmental exposures. Different researchers declared that several inherited risk factors assist in cardiovascular disease [41] showing that some of the risk genetic materials are shared from descendants to their generation [73]. These genetic polymorphisms prevail between black and white people, where their platelets react differently (hyper-reactive in black people) [42]. The point related to the historical observations reported that cardiovascular disease is highly developed in black people at a younger age, with the highest incidence of mortality compared with the white ones [74]. Thus, to identify the main reason for this variations in platelet reactivity; a study done on platelet RNA expression, discovered the existence of mRNAs and microRNAs correlated with individual’s race and PAR4 reactivity [6]. In 154 healthy individuals used during the study by Edelstein et al. (2013) [75], it was revealed that PAR4 activation induced the platelets to react differently. Their study showed that platelets from blacks express 14% more PAR4 protein than those from whites, but they reported that this difference is not associated with platelet PAR4 function. By using quantitative trait locus analysis their study discovered three common single nucleotide polymorphisms in the PAR4 gene which are associated with PAR4-induced platelet aggregation. Thereafter, they used one of them (rs773902) to determine the residue 120 in transmembrane domain 2 (checking if is alanine (Ala) or threonine (Thr). Interestingly, they discovered that Thr120 is more common in blacks than in white subjects at the level of 63% to 19%, respectively, and proved that it was associated with higher PAR4-induced human platelet aggregation and Ca^2+^ flux. Furthermore, Tourdot et al. (2014) [73], reported that platelets from black volunteers were hyperaggregable in response to PAR4-mediated platelet stimulation 2MeSAMP (2-methylthioadenosine 5′-monophosphate triethylammonium salt hydrate) compared with whites, which showed the highest level of activation through the Gq pathway compared with platelets from white donors, also demonstrated differences in Ca^2+^ mobilization, and integrin αIIbβ3 activation between black and white groups. Thus, according to this study, Gq pathway is differentially regulated by race in association to PAR4 activation [75].

The examination of platelet reactivity between white and black people from Philadelphia city also confirmed the hyper-reactivity of black platelets to the small concentration of PAR4 agonist compared to white people [74]. Additionally, it was also recognized that PAR4 sequence is differently mutated by polymorphisms at positions 120 of the PAR4 alleles for both races [22].

However, except to identify that platelets from black people express a high amount of PAR4 protein (over 14%) to that of white people [42], till now there is no defined mechanism explaining these PAR4 mutations between different races and how they are associated with activation. A possible assumption in this regard could be the high expression of PAR4 in black people which might be one of the reasons of their resistance to standard antiplatelet drugs leading to poorer cardiovascular treatment [71]. Another study on pharmacological effects of YD-3 antagonist showed that it inhibited PAR4-induced platelet activation in patients with 120A genotype but showed no effect on platelets from patients with 120T genotype [33].

## 17. Conclusions and Future Perspective

PAR1 and PAR4, in general, participate in vascular functions, and both of them are exposed receptors to be targeted by thrombin as well as other proteases that are responsible for blood coagulation. However, blocking of PAR1 and PAR4 agonists may be a solution to prevent tethered ligand formation; moreover, the inhibition of activation of these receptors might be important for further studies to achieve therapeutic progress against cardiovascular diseases.

Nowadays, lack of efficacious antiplatelet agents capable to effectively inhibit aggregation of platelets and thrombus formation without affecting normal hemostasis is still a big challenge. Although several PAR4 antagonists have been developed, still very few of them have been approved and tested in clinical trials. That is why, in this challenging field of study, more studies are required to understand the complex process of platelet signaling pathways, and all possible activation mechanisms, that will provide a great impact on the development of robust new drugs against thrombosis and other related diseases. It was further confirmed that in human platelets, not only PARs signal through thrombin receptors but also some other elements (like GPIb) assist independently in PAR response to thrombin. That is why designing of a complete inhibitor of thrombin-induced platelet activation could be better if it emphasizes the blockade to all types of thrombin receptors; however, it is not clear if those anti-PARs normally used are sufficient for anti-thrombotic effects able to inhibit these other elements for just optimizing outcomes in vivo.

In order to find and develop precisely selective inhibitors of PARs, further in-depth studies on platelet-dependent thrombus formation and its association with PARs with more focus towards cellular biology, biophysical cellular chemistry, and molecular genetics, are needed. Exodomain length of PAR4 is shorter as compared to PAR1, which may be a reason of difference in their cleavage sites. This difference of cleavage sites which associate with slower activation of PAR4 must be considered. Harmoniously, the dimerization mechanism between these two receptors could be also an important point to focus for novel drug development strategies because presently the drugs in practice are majorly focused to target PAR monomers.

Studies have reported that platelet reaction and receptor-thrombin interaction are not the same in different animal species, which might be one of the challenges for in vivo modeling leaving several unexplainable questions. Surprisingly, no precise endogenous proteinase has been reported to date for controlling the functions of PAR with in vivo models. Additionally, it is also not well understood whether synthetic TL PAR-AP (Tethered Ligand PAR-Activating Peptides) used in different studies, stimulate in the same way and through similar signaling cascade as that of proteases known to cleave the TLs.

Extensive studies have shown that some animal species possess different subfamily proteins of PAR like rabbit and rodents, which express PAR3 and PAR4, and guinea pigs which express PAR1 and PAR4 while rodents lackPAR1. Alternatively, it has been reported that humans possess PAR1 and PAR4, and it is known that the signal is solely through PAR4. This variation of difference of PAR family between human and rodents indicate a gap between preclinical (animal) and clinical (human) studies on homeostasis and thrombosis. A thorough exploration of these variations may be a further point to explore the alternative pathway of homeostasis and thrombosis in rodents and human that may be specifically through PAR4 with alternate knockdown of other PARs for better understanding. Some studies were successful in the exploration of the role of PAR3/PAR4 co-factoring in mouse platelets; however, it is yet to explore whether it can be applied to other animals in a similar fashion. Conclusively, a thorough experimental strategy with relevance to the species difference and its considerable implementation in humans needs a careful revision.

There is variation in platelets reactivity against PAR4 agonist between black and white individuals. As we know, Ca^2+^ mobilization enhances platelet activation, therefore, further in-depth studies are needed focusing more towards dissimilarity of Ca^2+^ mobilization among these racially different individuals. This dissimilarity of Ca^2+^ mobilization is associated with PAR4 activation so an increased expression of PAR4 in black people might be one of the reasons for increased resistance to standard antiplatelet drugs and poorer cardiovascular outcomes. This racial difference may provide a thought-provoking basis for further exploration in the direction to develop an effective antiplatelet drug that can be specified more towards the patients with African origin.

Further studies may be required to understand reliable proteases controlling the PAR activation in diseased conditions, especially in different types of cells in different pathological events. This may help for better understanding of PAR signaling pathways that may be associated with increased progression of cardiovascular diseases. As PARs have been identified in all vertebrates and in general their functions have also been explored to some extent, a study design focusing on the development of PAR4 antagonists or inhibitors can be a novel potential therapy in reducing cardiovascular diseases.

## Figures and Tables

**Table 1:** The PAR4 inhibitors, their targeting sites and some of their in vivo activities.

Inhibitor	Compound	Targeting Site	EC50	Reference	In Vivo Activity
Function Antibody	Rabbit Polyclonal	Thrombin Cleavage site	1 mg·mL^−1^	Wong et al., 2017 [37]	3.4 mg/kg in guinea pig showed that is an effective antithrombotic by lowering bleeding risk compared to clopidogrel [37]
Peptidomi-metics	tc-YPGKF-NH2	TLS binding site	100 μM	Hollenberg et al., 2004 [60]	Not reported
Low MW Compound	YD-3	TLS binding site	28 μM	Peng et al., 2004 [69]	10 mg/kg inhibited neointima development in rat [69]
	ML354	TLS binding site	140 nM	Wen et al., 2014 [43]	Not reported
	BMS-986120	TLS binding site	1 mg/kg	Wong PC et al., 2017 [37]	1 mg/kg decreased thrombus weight by 83% in monkey and increased BTs by about twofold compared to clopidogrel [37]
Pepducins	P4pal-10	Third intracellular loop	1 μM	French et al., 2016 [13]	3 μM inhibited ~85% of thrombin-induced aggregation of both human and mouse platelets, but also is an effective in vivo as it increased tail bleeding time in mice [13]
	P4pal-1	First intracellular loop	5 μmol·L^−1^	Leger et al., 2006 [36]	At low doses (0.13 mg/kg) decreased acute occlusion of carotid arteries in guinea pigs [36]
